# Supplementary material for: SOX11 and SOX4 drive the reactivation of an embryonic gene program during murine wound repair
Source: Nat Commun. 2019 Sep 6;10:4042. doi: 10.1038/s41467-019-11880-9 (PMC6731344; doi:10.1038/s41467-019-11880-9)
Supplement: Supplementary file 1 — Supplementary Information [file 41467_2019_11880_MOESM1_ESM.pdf]

SOX11 and SOX4 drive the reactivation of an embryonic gene program during murine wound repair

Miao et al.

Supplementary Information

Supplementary Figure 1

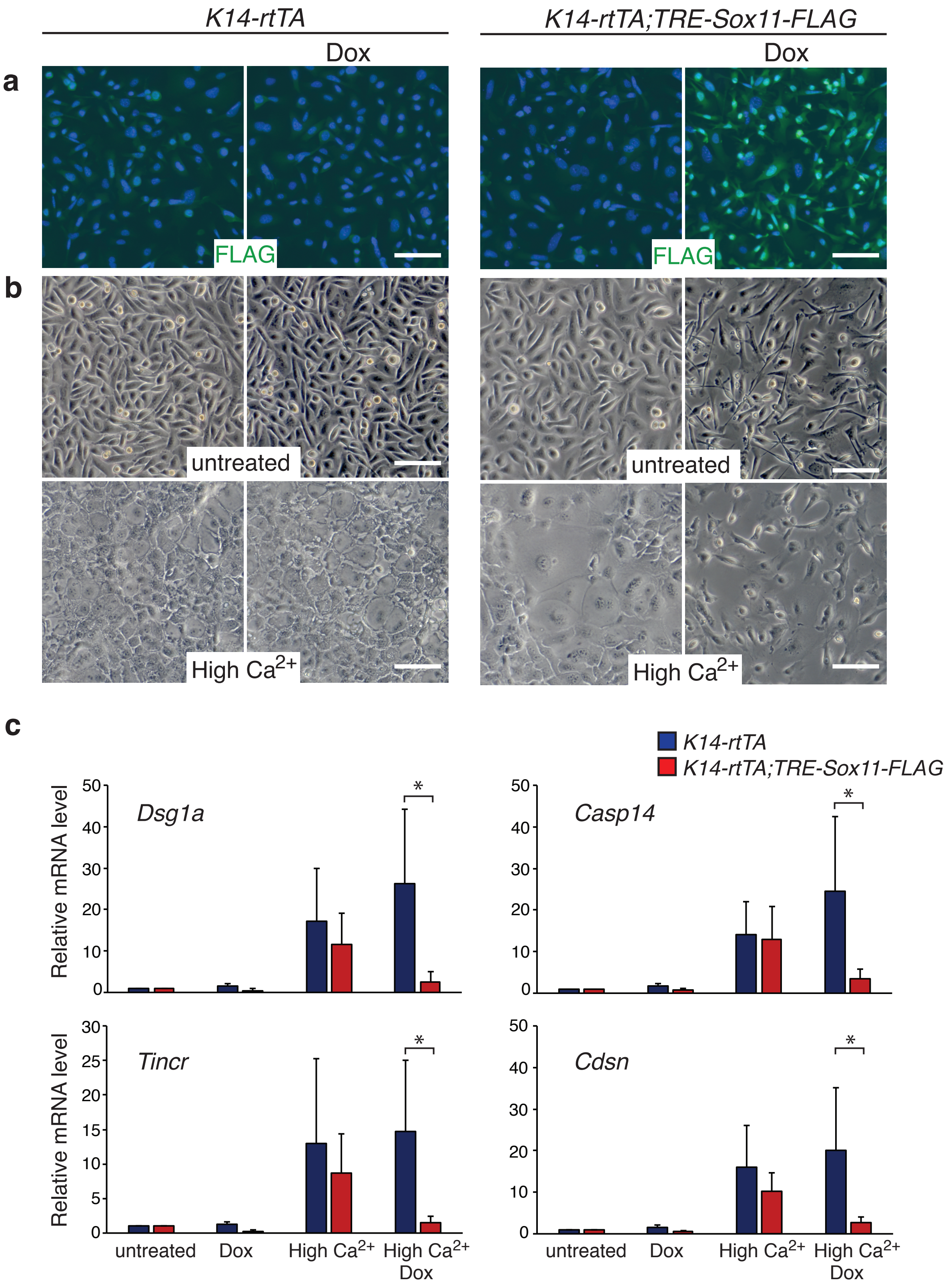

Supplementary Figure 1. SOX11 overexpression inhibits keratinocyte differentiation. (**a**) Immunofluorescence analysis of FLAG-tagged SOX11 induction in *K14-rtTA*;*TRE-Sox11-FLAG* keratinocytes *in vitro*. Primary keratinocytes from *K14-rtTA* (control) or *K14-rtTA*;*TRE-Sox11-FLAG* newborn skins were cultured with or without Dox (1 μg/ml) for 24 h prior to immunostaining for FLAG tag. (**b**) Effect of SOX11 expression on calcium-induced morphological changes of keratinocytes *in vitro*. Primary keratinocytes from *K14-rtTA* (control) or *K14-rtTA*;*TRE-Sox11-FLAG* newborn skins were cultured with or without Dox (1 μg/ml) 24 h prior to the addition of 1.5-mM calcium or vehicle control for an additional 24 h. (**c**) qRT-PCR analysis of epidermal differentiation gene expression in *K14-rtTA* (control) or *K14-rtTA*;*TRE- Sox11-FLAG* keratinocytes. Cells with or without Dox treatment were cultured with or without calcium (1.5 mM) for 24 h. The gene expression level in the *K14-rtTA* cells without any treatment was set as 1. Data are the mean ± SD. $n = 5$ biologically independent samples. *$p<0.05$ (Student's one-tailed *t*-test). Scale bar, 100 μm. Images in panels **a** and **b** are representative of images from 2 and 3 experiments, respectively. The selected images reflect the images detected uniformly across the plate of each sample. Source data for panel **c** are provided as a Source Data file.

Supplementary Figure 2

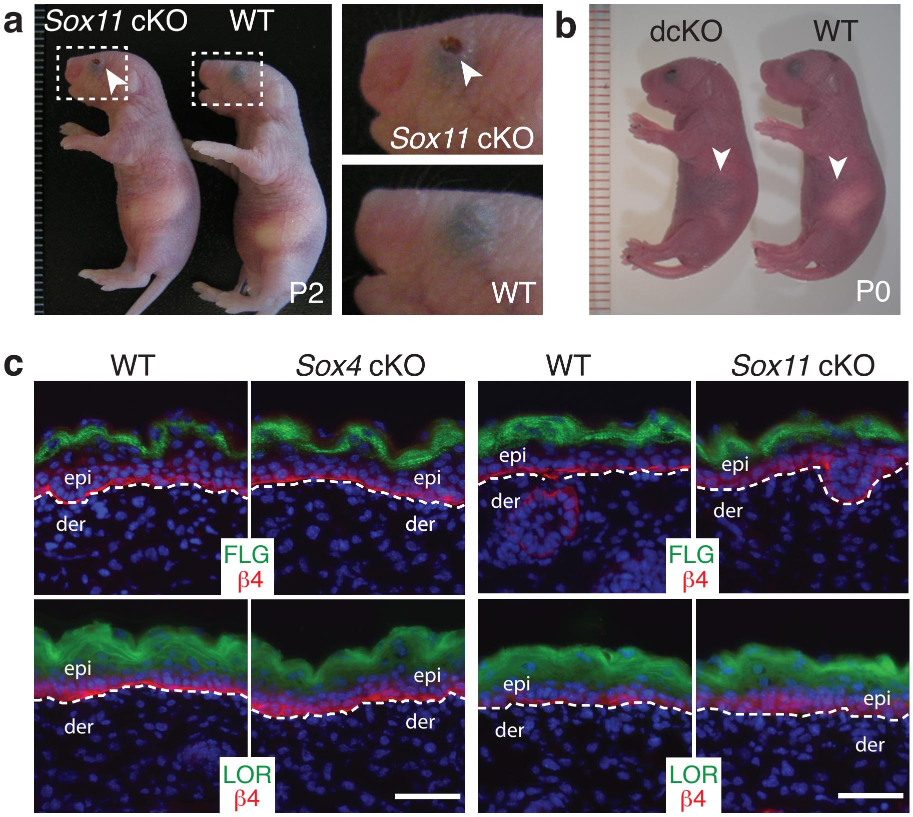

Supplementary Figure 2. Effects of deficiency of *Sox11* and *Sox4*. (**a**) Images of WT and *Sox11* cKO neonates. Note the open eyes in *Sox11* cKO pups (arrow-headed). (**b**) Images of WT and newborns deficient of both *Sox11* and *Sox4.* Note the lack of milk spot (arrow-headed) in the dcKO. (**c**) Immunofluorescence analysis of epidermal differentiation markers in embryos lacking either *Sox11* or *Sox4* at E16.5. White dashed lines demark the epidermal and dermal border. Scale bar, 50 μm. Neonates in the images in panels **a** and **b** are representative of those from all (>20) the litters observed with 8-12 animals each litter. Images in panel **c** are representative of images from 2 experiments on 2 different pairs of animals. The selected images reflect the images detected uniformly across the skin section of each sample.

Supplementary Figure 3

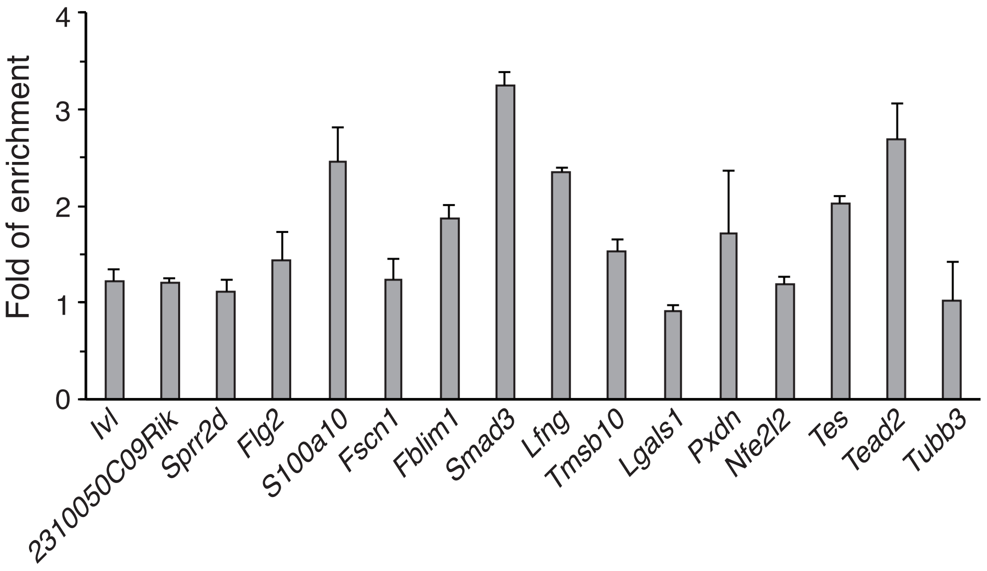

Supplementary Figure 3. ChIP-qPCR analysis of epidermal cells over-expressing SOX11. ChIP was performed with anti-FLAG antibody on ~10 million pooled epidermal basal cells isolated from *K14rtTA;TRE-Sox11-FLAG* 4-day old pups 24 h after Dox treatment. qPCR analysis was done using primers spanning regions containing SOX4/11- binding motif or regions without the conserved binding sites (negative control) of selected targets identified by ChIP-seq. The graph shows the relative amount of the region containing SOX11/4 binding motif bound to SOX11-FLAG relative to the negative control region. Data are mean ± SD. n = 2 biologically independent samples. Source data are provided as a Source Data file.

Supplementary Figure 4

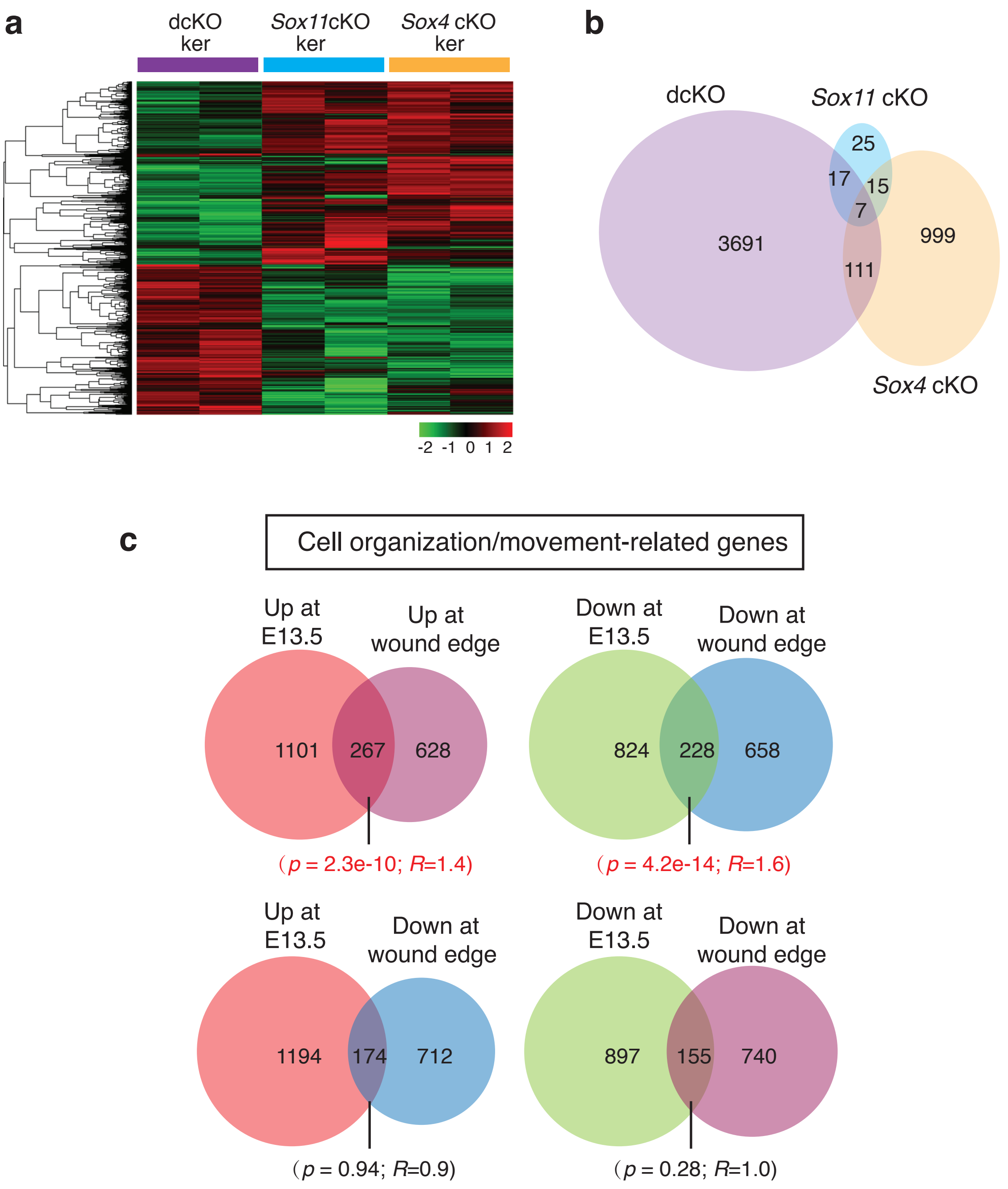

Supplementary Figure 4. Transcriptomic profiling of primary keratinocytes deficient of both *Sox11* and *Sox4* or of either gene alone. (**a**) Primary keratinocytes cultured from newborn littermates of indicated genotypes were subjected to the two-color microarray analysis with 2 biological replicates per genotype. Heat map shows clustering of genes with significant differential expression (log2-fold change >1.5 and FDR<0.05) in dcKO, *Sox11* cKO or *Sox4* cKO primary keratinocytes relative to their wild-type controls. (**b**) Venn diagram showing the overlap of the probesets significantly changed in dcKO, *Sox11* cKO or *Sox4* cKO keratinocytes. (**c**) Venn diagrams comparing cell organization and cell migration related genes that are differentially expressed in epidermal cells at E13.5 versus P4 and at wound edge versus unwounded[1]. Statistical significance (highlighted in red) is found in the overlap between genes upregulated at E13.5 and at the wound edge (p = 2.3e-10, R>1) and between genes downregulated at E13.5 and at the wound edge (p = 4.2e-14, R>1). Source data for the lists of genes are provided as a Source Data file.

Supplementary Figure 5

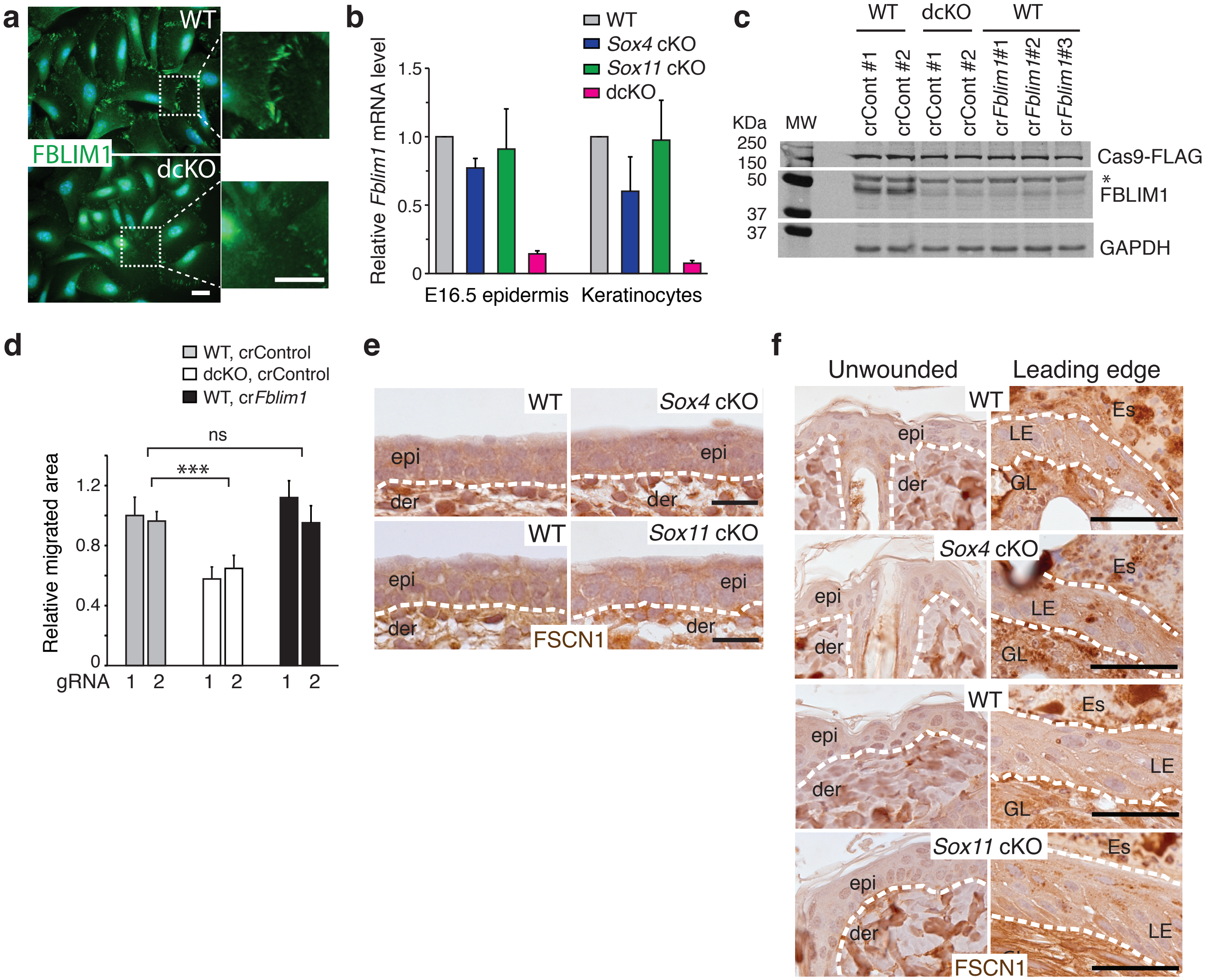

Supplementary Figure 5. Regulation of *Fblim1* and *Fscn1* by SOX11 and SOX4. (**a**) Immunofluorescence analysis of FBLIM1 expression in WT and dcKO primary keratinocytes. Images are representative of images from 2 independent experiments. (**b**) RT-qPCR analysis of *Fblim1* expression in isolated E16.5 epidermis and primary keratinocytes of specified genotypes. Data are the mean ± SD. *n* = 2 (*Sox11* cKO-control pairs), or 3 (*Sox4* cKO-control pairs, and dcKO-control pairs) biologically independent samples. (**c**) Western blot analysis of CRISPR/Cas9-mediated *Fblim1* knockout. WT or dcKO primary keratinocytes were transduced with lentiviral vectors expressing FLAG tagged CRISPR/Cas9 and control gRNAs or gRNAs targeting *Fblim1*. *, non-specific bands. The Western blot image is available in Supplementary Figure 6. (**d**) Effect of ablation of *Fblim1* on migration. Graph quantifying relative areas the cells migrated normalized over the controls (WT cells expressing control gRNAs). Data are the mean ± SD. *n* = 3 independent experiments, with 16-18 fields quantified per replicate. ***p<0.001, ns, not significant (Student's two-tailed *t*-test). (**e**) Immunochemical analysis of FSCN1 expression in E14.5 skin deficient of either *Sox11* or *Sox4*. (**f**) Immunohistochemical analysis of FSCN1 expression at wound edge of *Sox11- or Sox4*-ablated and control skins 5 days post wounding. Epi, epidermis; Der, dermis; Es, eschar; LE, leading edge; GL, granulation layer. Scale bars, 20 μm (**a, e**), 50 μm (**f**). Images in panels **e** and **f** are representative of images from 3 experiments, reflecting the images detected through the plate of each sample (**a**), or the entire skin section of each sample (**e** and **f**). Source data for panel **b-d** are provided as a Source Data file.

Supplementary Figure 6

a Fig. 6a

b Supplementary Figure 5c

c Fig. 9i

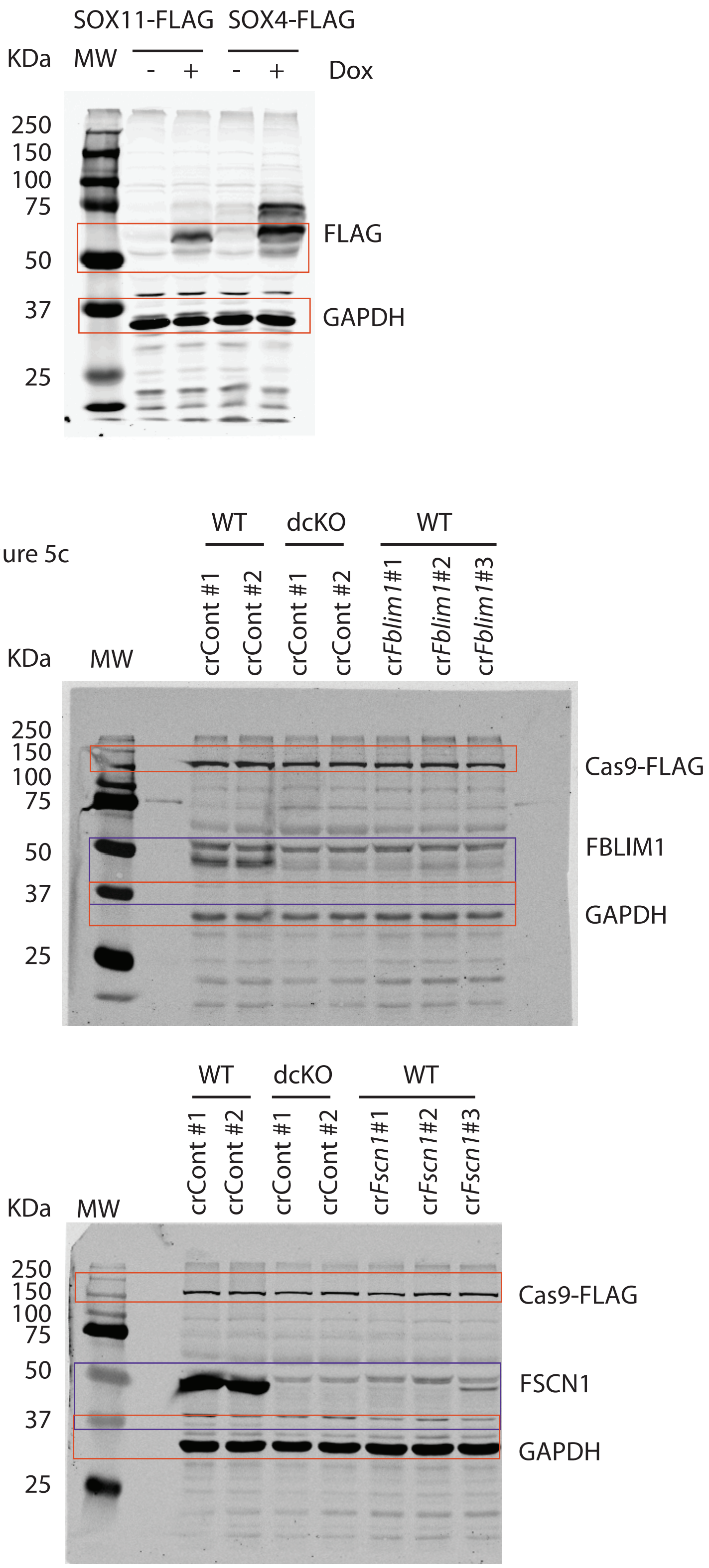

Supplementary Figure 6. Unprocessed images of Western blots. Uncropped Western blot images of scanned immunoblots shown in **Fig. 6** (**a**), **Supplementary Figure 5** (**b**), and **Fig. 9** (**c**).

Supplementary Table 1

Gene ID	Gene Symbol	E13.5 *vs* P4	WdEpi*
11568	*Aebp1*	3.34	3.36
100182	*Akna*	3.24	3.87
11792	*Apex1*	1.53	1.52
70350	*Basp1*	4.85	11.42
53314	*Batf*	2.81	7.97
381319	*Batf3*	2.21	7.48
320705	*Bend6*	4.40	5.33
66653	*Brf2*	1.51	1.52
12398	*Cbfa2t3*	1.92	7.83
13395	*Dlx5*	2.24	2.01
18612	*Etv4*	2.09	2.78
14247	*Fli1*	1.88	8.50
57441	*Gmnn*	1.52	1.94
56198	*Heyl*	1.51	5.53
16909	*Lmo2*	1.82	5.93
94352	*Loxl2*	3.78	6.77
17095	*Lyl1*	3.20	3.52
17865	*Mybl2*	1.91	2.29
18102	*Nme1*	1.87	2.71
231602	*P2rx2*	4.88	3.82
18511	*Pax9*	1.86	5.14
59093	*Pcbp3*	2.17	2.55
18740	*Pitx1*	9.05	6.34
320795	*Pkn1*	6.70	3.26
18751	*Prkcb*	2.45	4.01
18933	*Prrx1*	3.15	8.29
19663	*Rbpms*	4.09	5.88
104383	*Rcor2*	2.09	1.80
20666	*Sox11*	13.68	6.10
22160	*Twist1*	3.59	7.06
13345	*Twist2*	14.06	6.35
21417	*Zeb1*	5.08	4.42

Supplementary Table 1. Relative expression level of transcription (co)factors upregulated in epidermal cells at E13.5 and the wound edge. The table shows the log2-fold change of the transcription factors and co-factors that are upregulated in epidermal cells at E13.5 (versus P4) and at the wound edge (WdEpi, versus unwounded). *, data from Ge *et al*. (2017)[1].

Supplementary Table 2

Cornified envelope genes increased in dcKO
Lelp1 (4.5)<br><br>*Sprr* family: ***2a2*** (3.9), ***2d*** (3.2), *2h* (2.3), *2k* (1.6), ***1b*** (1.8), ***Ivl*** (2.6)<br><br>*Lce* family:*1a1* (4.7),***1b*** (24.7),*1a2* (9.4),*1c* (13.2), *1d* (6.7),*1e* (5.5),*1f* (5.9),*1g* (8.4),*1h* (14),*1i* (8.5), *1j* (7.2), *1k* (17.5), *Kprp* (9.5), *1l* (11.1), ***2310050C09Rik*** (20), *3b* (1.8), *3c* (1.9), *3d* (1.9), *3e* (1.8), *Crct1* (2.2), ***1m*** (18.1)<br><br>***Flg2*** (7.4), *Flg* (25.3), *Hrnr* (1.6), ***Rptn*** (1.8)

Supplementary Table 2. The cornified envelope genes are highly induced in dcKO epidermis at E16.5. The genes encoding cornified envelope proteins are located in the epidermal differentiation cluster (EDC) on mouse chromosome 3 (see **Fig. 7d**). The table shows the fold-changes of the differentially expressed EDC genes (FDR<0.05 and fold change>1.5, log2-fold change). The genes in bold font are bound by SOX11 and/or SOX4.

Supplementary Table 3

Gene ID	Gene Symbol	E13.5 *vs* P4	WdEpi*	dcKO E16.5 epi	dcKO keratinocytes	Cell Organization /Movement
16800	*Arhgef2*	2.31	1.81	-1.80	-2.13	Y
18612	*ETV4*	2.09	2.78	-1.65	-6.13	Y
14026	*Evl*	2.20	3.82	-2.11	-12.20	Y
223254	*Farp1*	1.63	2.11	-1.64	-4.20	Y
74202	*Fblim1*	5.22	8.25	-2.12	-7.25	Y
14086	*Fscn1*	2.53	6.20	-1.84	-7.94	Y
381633	*Gm1673*	15.49	3.80	-1.77	-2.29	N
14702	*Gng2*	4.33	5.27	-1.56	-8.77	N
224023	*Klhl22*	4.60	4.20	-1.80	-12.05	Y
16852	*Lgals1*	4.21	7.70	-2.18	-76.92	Y
67803	*Limd2*	5.36	3.02	-2.51	-3.48	N
17357	*Marcksl1*	5.52	1.96	-2.26	-5.99	N
18542	*Pcolce*	11.40	2.08	-3.32	-2.06	N
69675	*Pxdn*	6.70	1.71	-1.66	-41.67	Y
104383	*Rcor2*	2.09	1.80	-1.85	-3.09	N
26564	*Ror2*	3.71	3.74	-1.55	-8.20	Y
109232	*Sccpdh*	2.24	1.72	-1.83	-2.30	N
20317	*Serpinf1*	10.11	7.25	-2.08	-2.12	Y
218756	*Slc4a7*	2.29	1.72	-1.66	-2.46	N
20621	*Snn*	3.55	2.66	-1.82	-2.56	N
68875	*Tmcc2*	2.18	2.11	-1.60	-1.52	N
230157	*Tmeff1*	4.32	1.68	-1.72	-1.72	N
19240	*Tmsb10*	2.75	3.46	-2.45	-2.78	Y
13345	*Twist2*	14.06	6.35	-2.98	-7.14	N
13003	*Vcan*	4.62	3.33	-2.93	-15.63	Y
100072	*Camta1*	-5.92	-2.43	1.57	2.64	N
71884	*Chit1*	-13.89	-1.52	1.59	1.53	N
19419	*Rasgrp1*	-9.35	-3.52	1.54	7.49	Y
108116	*Slco3a1*	-6.62	-1.96	1.55	2.69	N

Supplementary Table 3. Relative expression level of genes up- or down-regulated in epidermal cells at E13.5 and the wound edge directly regulated by SOX11 and SOX4. The table shows the log2-fold of the differentially expressed genes that show direct binding to SOX11 and/or SOX4 and are overlapped between specified transcriptomes: epidermal development (E13.5 *vs* P4), E16.5 epidermis (dcKO E16.5 epi) or primary keratinocytes (dcKO keratinocytes), and wound edge (WdEpi). Genes are categorized as functionally involved in the biological processes related to cell organization/movement (Y) or not (N). *, data from Ge *et al.* (2017)[1].

Supplementary Table 4

Supplementary Table 4. Sequences of oligos

Primers for Cloning

*Sox11*_F_*Xho*I	gac c tcgagc ATGGTGCAGCAGGCCGAGAGCTC	To pcDNA3.1	
*Sox11*_R_*Sfu*I	aga ttcgaa ATACGTGAACACCAGGTCGGAGA		
*Sox4*_F_*Xho*I	gac c tcgagc ATGGTACAACAGACCAACAACG	To pcDNA3.1	
*Sox4*_R_*Apa*I	ata gggccc GCGTAGGTGAAGACCAGGTTAGAGATG		
*Sox4*_F_*Hind*III	gac aagctt ACC ATGGTACAACAGACCAACAACG	To pENTR 1A	
*Sox4*_R_*Eco*RV	ata gatatc GTAGGTGAAGACCAGGTTAGAGATG		
*Fscn1*_F_*Hind*III	gac aagctt ACC ATGACCGCCAACGGCACGGC	To pcDNA3.1	
*Fscn1*_R_*Eco*RV	ata gatatc GTACTCCCAGAGTGAGGCGGGGTC		
*Tead2*_enh_*Sal*I_F	tta gtcgac TTCTATCTGGGAGTCTACTCCCTTC	To pGL3-TATA	chr7: 52470841-52472571, 1731 bp
*Tead2*_enh_*Sal*I_R	tta gtcgac AAGATCAGCAGCTACATATGGTATTGC		
*Fscn1*_enh_*BamH*I_F	ata ggatcc CAGGATCGGGGTAGTAGATTATAAAG	To pGL3-TATA	chr5:143724688-143726687, 2000 bp
*Fscn1*_enh_*BamH*I_R	ata ggatcc ACCATCAAGTGGAGGCCAGAAG		
*Fblim1*_enh1_*Xho*I_F	ata ctcgag GGAGGGCAAATCAGACACCCAG	To pGL3-TATA	chr4:141154621-141156000, 1380 bp
*Fblim1*_enh1_*Xho*I_R	tct ctcgag GGTGCACCGTGGGGGTGGGCAG		
*Fblim1*_enh2_*Xho*I_F	ata ctcgag CTTTATATCCGTTCCAGACGGG	To pGL3-TATA	chr4:141130909-141131511, 603bp
*Fblim1*_enh2_*Xho*I_R	tct ctcgag GCGTTCAGCCGCTAGGGGGC		
*Marcksl1*_enh_*Xho*I_F	ata ctcgag GCTCCCCGGGGCGACGTGACC	To pGL3-TATA	chr4:129191095-129191672, 578bp
*Marcksl1*_enh_*Xho*I_R	tct ctcgag AGCACTACTGGGCGGGAAGCAG		
*Pxdn*_enh_*BamH*I_F	ata ggatcc ACCACCGTGCGCTGCATGCATCTG	To pGL3-TATA	chr12:30623122-30623658, 537bp
*Pxdn*_enh_*BamH*I_R	ata ggatcc TGATGTGGGGTGGGCATCCTTCC		

Primers for real-time PCR

	**Forward**	**Reverse**
Mrpl19	AGAGGCAGGAGGGTTCCAAG	GGGCTTCATGAGACCACGAC
Hprt	CAGGCCAGACTTTGTTGGATT	TTGCGCTCATCTTAGGCTTT
Sox4	GATGCGTTTGGCATTTGTGT	TCTCCAGCTGCAAGGACAAG
Sox11	TTGGTGTCTCAGCATCCAACCAG	AGCCTGCCCTAAGCATCACTTC
Sox12	TCAGTTCTTCCTCGGCGCATTC	ACATTCACTGGACAAGGCAACG
Krt1	GGACATGGAGATTGCCACA	CTACTGCTTCCGCTCATGCT
Krt5	ATGAACCGAATGATCCAGAGG	AATGGCGTTCTGGAGGTTG
Dsg1a	AAGGGGATCCTGATGAAACC	CACGTGTGAATTGCTCCATC
Casp14	CCAACCTATACGGATACCCTCC	AGTCGGGTGATCTCTTCTGTC
Cdsn	CTGATGGCCGGTCTTATTCT	GCTGTTGGAGCCAGTCTTTC
Ivl	CCTGTGAGTTTGTTTGGTCTACA	GGATGTGGAGTTGGTTGCTT
Tincr	TGCCTGACCATCAGACAGTTC	TCCTTCAGCCAGCATCTTGT
Flg	GCAAGTGGTCAGGGAGGATAT	GGAACGATATACCTGGAGATGC
Lor	TCACTCATCTTCCCTGGTGCTT	GTCTTTCCACAACCCACAGGA
Lce1a	CACTTTAGACAAACCATTCAGGAGAA	CCAAGAAGACAAACCCAGCAA
Fblim1	TCTGCGAGAATCCCATCATC	CGGTAGGTTCCACAGACAGG
Fscn1	GGCGCCTACAACATCAAAGAC	CCACCTTGAGAGCCACCTT

Primers for genotyping

	**Forward**	**Reverse**	**Product size**
TRE-Sox11-3×FLAG	AGGACCTGGATTCCTTCAGC	AACTCACTTGTCATCGTCATCCT	212 bp

Primers for ChIP-qPCR

	**Forward**	**Reverse**
Tead2	ATGCCTGGGCTCTTTGTTCT	TGGGTGCCCATCAAACTTC
*Tead2*_negative site	CAACTTGTGGTGCATTCTGG	CAGTCAAACTCCCTGCTTCTG
Tubb3	GGGCCTGGGTTCTATTGTCC	CCCTCGCTGGCTGATGTAAG
*Tubb3*_negative site	TCAGCCTTGGTGACATAGGAA	GCTGGACGCCAAGTCTCTAC
Ivl	GGAAACCATGACTAAGCCTCTG	ACAAGAATGGTGTCAGTCAACAAC
2310050C09Rik	AGTCACGTGTCAGCCTCCA	GGCAGGTCCAGAAATCCAG
Sprr2d	CCCAGATGCAGAACAGATGACT	GGCAGGTCCAGAAATCCAG
Flg2	TTGGTAAATGTAAGGGTGTGGTC	GCCTGGGTTAGAAATCTCTTGTG
S100a10	CCAGGAAAGCCAGACACATT	CCACACCCAAGGTCTCACA
*EDC*_Negative site 1	GAAGGACAGGCAAGAAAGAAGGT	CGCAGCTCTTTCAAGTGGTAATCT
*EDC*_Negative site 2	ACAAGAGCATGGCCTGTACCA	TACCCAGCTCCTTCTACTGCTTTC
*EDC*_Negative site 3	CCAAAGCTCCTATGCTTCCA	GTTCCACTGTTGGCTTTGCT
Fscn1	GCCTTGGTGGCCTCTTTATT	CCAACCTTCAACCTCCCAGT
Fblim1	GGAGGGCAAATCAGACACC	TCAGCCTCCTTCCTTCCTCT
Smad3	TTGTGGAGAAGGGCCAAA	CGGCTCTCTCTCTAACCACCT
Lfng	AGGCCAGCTGTATGGAAATG	CAGACAAAGGGACCCGAAG
Tmsb10	ACCCAGCCGAGGTAAGTTG	CCCAGCAGGAGATTCCATT
Lgals1	GGCCAGGTTCGCAGATTTAG	CGACTAGACCCAGCGAGGAA
Pxdn	GGGTACTGGTTTGGAGAGGTG	CGGGAGATTCCGAGTTTGA
Tes	CACTCCTCTTGGCCTGCTT	CTTGCACCCACTGGACTTCT
Nfe2l2	CACAAGTCTGTTAACCCAAAGCA	GGTGTAAGTCATGCGCCAGT
Negative_1	CACAGTTCCAGGGTTCCAGT	CTGCTCCTCACTGTCCTGCT
Negative_2	GGAGGCTGGCAGAGCAGTC	AGCCCTTCACAGTCCCTCCTA
Negative_3	CAAACAGCTAAGCCCAGGAA	GCAGCAGCAGCACAGAAA
Negative_4	TAGCTGGAAAGCCCAAATCA	ATCCATGCTCCCTTCTTGGA

Oligos for gRNA cloning

	**Forward**	**Reverse**
Fscn1, pair 1	caccg GTAGACCGCGACGTGCCTTG	aaac CAAGGCACGTCGCGGTCTAC c
Fscn1, pair 2	caccg TGCGAGCGCGAGGTGCCCGA	aaac TCGGGCACCTCGCGCTCGCA c
Fscn1, pair 3	caccg GCTACGCGCATCTGAGCGCG	aaac CGCGCTCAGATGCGCGTAGC c
Fblim1, pair 1	caccg AGGAGCCTCCTGTCTTACCA	aaac TGGTAAGACAGGAGGCTCCT c
Fblim1, pair 2	caccg GAGGCATTGGTCTTGCCAGA	aaac TCTGGCAAGACCAATGCCTC c
Fblim1, pair 3	caccg TTTGTGGGAAGCATCTCCCA	aaac TGGGAGATGCTTCCCACAAA c
Control, pair 1	caccg GCACTACCAGAGCTAACTCA	aaac TGAGTTAGCTCTGGTAGTGC c
Control, pair 2	caccg GTCTCCACGCGCAGTACATT	aaac AATGTACTGCGCGTGGAGAC c

SUPPLEMENTARY REFERENCES

1. Ge, Y. *et al.* Stem cell lineage infidelity drives wound repair and cancer. *Cell* **169**, 636-650 e614 (2017).
