# Supplementary material for: SOX11 and SOX4 drive the reactivation of an embryonic gene program during murine wound repair
Source: Nat Commun. 2019 Sep 6;10:4042. doi: 10.1038/s41467-019-11880-9 (PMC6731344; doi:10.1038/s41467-019-11880-9)
Supplement: Supplementary file 3 — Reporting Summary [file 41467_2019_11880_MOESM3_ESM.pdf]

nature research

Corresponding author(s): Hoang Nguyen

Reporting Summary

Nature Research wishes to improve the reproducibility of the work that we publish. This form provides structure for consistency and transparency in reporting. For further information on Nature Research policies, see Authors & Referees and the Editorial Policy Checklist.

Statistical parameters

When statistical analyses are reported, confirm that the following items are present in the relevant location (e.g. figure legend, table legend, main text, or Methods section).

n/a	Confirmed	
☐	☒	The exact sample size (n) for each experimental group/condition, given as a discrete number and unit of measurement
☐	☒	An indication of whether measurements were taken from distinct samples or whether the same sample was measured repeatedly
☐	☒	The statistical test(s) used AND whether they are one- or two-sided<br>*Only common tests should be described solely by name; describe more complex techniques in the Methods section.*
☐	☒	A description of all covariates tested
☒	☐	A description of any assumptions or corrections, such as tests of normality and adjustment for multiple comparisons
☐	☒	A full description of the statistics including central tendency (e.g. means) or other basic estimates (e.g. regression coefficient) AND variation (e.g. standard deviation) or associated estimates of uncertainty (e.g. confidence intervals)
☐	☒	For null hypothesis testing, the test statistic (e.g. F, t, r) with confidence intervals, effect sizes, degrees of freedom and P value noted<br>*Give P values as exact values whenever suitable.*
☒	☐	For Bayesian analysis, information on the choice of priors and Markov chain Monte Carlo settings
☒	☐	For hierarchical and complex designs, identification of the appropriate level for tests and full reporting of outcomes
☒	☐	Estimates of effect sizes (e.g. Cohen's d, Pearson's r), indicating how they were calculated
☐	☒	Clearly defined error bars<br>*State explicitly what error bars represent (e.g. SD, SE, CI)*

Our web collection on statistics for biologists may be useful.

Software and code

Policy information about availability of computer code

Data collection	The microarray slides were scanned with the Agilent Scanner (G2565BA) using Scanner Version C and Scan Control software version A.8.5.1. Data extraction and quality assessment of the microarray data was completed using Agilent Feature Extraction Software Version 11.0.1.1.
Data analysis	GSEA v3.0 [build: 0160] was used in the gene set enrichment analysis.<br>ChIP-seq reads were mapped to the mouse genome (mm9) using Bowtie2 v2.3. Peak calling and Motif enrichment analysis were carried out with Homer v4.9 (findPeaks -style factor) and (findMotifsGenome.pl), respectively.<br>All gene ontology (GO) enrichment analysis was performed using Metascape v3.0.<br>The Bioconductor package limma v3.5 was used to analyze the microarray data.<br>ImageJ v1.51 was applied to determie the migrated areas in the in vitro wound healing assays.<br>R v3.4.3 and the gmp package were used to calculate the Venn diagram hypergeometric p values.

For manuscripts utilizing custom algorithms or software that are central to the research but not yet described in published literature, software must be made available to editors/reviewers upon request. We strongly encourage code deposition in a community repository (e.g. GitHub). See the Nature Research guidelines for submitting code & software for further information.

Data

Policy information about availability of data

All manuscripts must include a data availability statement. This statement should provide the following information, where applicable:

- Accession codes, unique identifiers, or web links for publicly available datasets
- A list of figures that have associated raw data
- A description of any restrictions on data availability

Microarray and ChIP-seq data from this study have been deposited in the Gene Expression Omnibus (GEO) under accession codes GSE120827 (transcriptional profiles of Sox4cKO, Sox11cKO, and Sox4/11 dcKO mouse keratinocytes and their wild-type control), GSE120826 (transcriptional profiles of Sox4 cKO, Sox11 cKO, and Sox4/11 dcKO mouse epidermis at E16), GSE120824 (transcriptional profiles of murine E13 epidermal cells and P4 epidermal basal cells), GSE120825 (transcriptional profile of SOX11-induced mouse epidermis), and GSE120773 (ChIP-seq) respectively. The source data underlying Figs 1a-c, 3a,c,d, 4a, d-f, 5a-f, 6a,b,h,j, 7a, 8b,c,f,g, 9d,h-j,l and Supplementary Figs 1c, 3, 4a-c and 5b-d are provided as a Source Data file. All other data supporting the findings of this study are available from the corresponding author on reasonable request.

Field-specific reporting

Please select the best fit for your research. If you are not sure, read the appropriate sections before making your selection.

☒ Life sciences ☐ Behavioural & social sciences ☐ Ecological, evolutionary & environmental sciences

For a reference copy of the document with all sections, see nature.com/authors/policies/ReportingSummary-flat.pdf

Life sciences study design

All studies must disclose on these points even when the disclosure is negative.

Sample size	No sample-size calculation was performed. Sample sizes were chosen based on experiments from published literature.
Data exclusions	No exclusion
Replication	Because of variability in the fertilization, implantation and development, it is difficult to predict developmental stage, even using a 6h mating window. To achieve precise developmental stage, multiple mice were mated for each gestational age. Several litters for each embryonic age were obtained which developmental stage was verified by morphological and histological analysis. In the X-gal exclusion assay, because the skin barrier develops very quickly at E16-17, it might be too early or too late to observe the difference in the epidermal differentiation. To ensure the replicability, we analyzed multiple litters from each mating pair, genotyped all the embryos from each litter, and used the litters in which the wild-type embryos started to acquire the barrier at the dorsal initiation sites (negative by permeability assay).<br>After many trials, we had maximized the replicability in the reepithelization assays in our settings. We used the mold to outline the pattern for the wounds to be made. Particular attention was paid to secure the intactness of the splints till the wounds were harvested. We discarded any samples with the wound beds destroyed during the whole procedures.<br>Biological replicates were used in all experiments and the findings were reproducible.
Randomization	Age-matched mice in experimental groups were selected based on their genotypes.<br>Other samples were not grouped and hence no randomization was performed.
Blinding	Re-epithelialization measurement was done by a staff member who was blinded to group allocation.

Reporting for specific materials, systems and methods

Materials & experimental systems

n/a	Involved in the study
☐	☒ Unique biological materials
☐	☒ Antibodies
☒	☐ Eukaryotic cell lines
☒	☐ Palaeontology
☐	☒ Animals and other organisms
☒	☐ Human research participants

Methods

n/a	Involved in the study
☐	☒ ChIP-seq
☐	☒ Flow cytometry
☒	☐ MRI-based neuroimaging

Unique biological materials

Policy information about availability of materials

Obtaining unique materials	all lab generated unique biological materials will be available upon request.

Antibodies

Antibodies used	Anti-SOX11, rabbit polyclonal, Sigma HPA000536; RRID: AB_1080060.<br>Anti-SOX11, guinea pig polyclonal, Elisabeth Sock and Michael Wegner laboratory, gpSox11; RRID:AB_2722601.<br>Anti-BrdU, rat monoclonal, Abcam ab6326; RRID:AB_305426.<br>Anti-CD104, rat monoclonal, BD Biosciences 553745; RRID:AB_395027.<br>Anti-KRT5, rabbit polyclonal, BioLegend 905501; RRID:AB_2565050.<br>Anti-FLG, rabbit polyclonal, BioLegend 905801; RRID:AB_2565053.<br>Anti-LOR, rabbit polyclonal, BioLegend 905101; RRID:AB_2565046.<br>Anti- DYKDDDDK Tag, rabbit polyclonal, Cell Signaling 2368; RRID: AB_2217020.<br>Anti-TCF7L2, rabbit monoclonal, Cell Signaling 2569S; RRID:AB_2199816.<br>Anti-KRT18, mouse monoclonal, Cell Signaling Tech 4546; RRID: AB_2134843.<br>Anti-FSCN1, mouse monoclonal Santa Cruz Biotechnology sc-21743; RRID:AB_627580.<br>Anti-FBLIM1, mouse monoclonal Santa Cruz Biotechnology sc-271417; RRID:AB_10610507.<br>Anti-VCL, mouse monoclonal Novus Biologicals NB600-1293; RRID:AB_2272814.<br>Anti-KRT1, rabbit polyclonal Fuchs lab.<br>Anti-TCF7L1, guinea pig polyclonal Hoang Nguyen lab.<br>ANTI-FLAG M2, mouse monoclonal Sigma F1804; RRID:AB_262044.<br>Anti-GAPDH, rabbit polyclonal Bethyl A300-641A; RRID:AB_513619.
Validation	All commercially available antibodies, listed with catalogue number and identifier, have validation statements on the manufacturer's website.<br>See the references associated with each RRID.<br>Anti-SOX11 (Sigma HPA000536, Elisabeth Sock and Michael Wegner laboratory): the positive immunostainings were verified by qPCR analysis, or with Sox11cKO skin.<br>Lab generated antibodies (Anti-KRT1, Anti-TCF7L1, Anti-SOX11) have been validated in citations listed below.<br>Guinea pig polyclonal anti-TCF7L1, Nguyen Lab:<br>Miao, Q., Nishino, Y., Ku A., Howard, J.M., Rao, A.S. Shaver, T.M., Garcia, G.E., Le D.N., Karlin, K.L., Westbrook, T.F., Poli, V., and Nguyen, H. (2014) Nature Communications 5:4088. doi:10.1038/ncomms 5088. PMID: 24909826; PMCID: PMC4052366.<br>Guinea pig polyclonal, anti-SOX11, Sock and Wegner:<br>Koch JM, Bösl MR, Wegner M, Sock E. (2008) Mol Cell Biol. 28:4675-4687

Animals and other organisms

Policy information about studies involving animals; ARRIVE guidelines recommended for reporting animal research

Laboratory animals	Mouse. Transgenic: K14-rtTA, K14-Cre (FVB/N), Krt14-H2BGFP (FVB/N), Sox4fl/fl;Sox11fl/fl;Sox12–/– (129SvEx:C57BL/6 mixed background), TRE-Sox11-FLAG (FVB/N), K14-rtTA;TRE-Sox11-FLAG (FVB/N), K14-Cre; Sox4fl/fl, K14-Cre; Sox11fl/fl, Krt14-Cre;Sox4fl/fl;Sox11fl/+, Krt14-cre;Sox4fl/+;Sox11fl/fl, Krt14-cre;Sox4fl/fl;Sox11fl/fl, Krt14-cre;Sox4fl/fl;Sox11fl/fl;Sox12–/-; BALB/c Nude Mouse; FVB/N
Wild animals	n/a
Field-collected samples	n/a

ChIP-seq

Data deposition

☒ Confirm that both raw and final processed data have been deposited in a public database such as GEO.

☒ Confirm that you have deposited or provided access to graph files (e.g. BED files) for the called peaks.

Data access links<br>*May remain private before publication.*	https://genome.ucsc.edu/cgi-bin/hgTracks?hgS_doOtherUser=submit&hgS_otherUserName=mchill&hgS_otherUserSessionName=Qi_Miao_Sox4_Sox11_ChIPseq
Files in database submission	bedgraphs, peak files, raw sequencing fastq files.
Genome browser session<br>(e.g. UCSC)	https://genome.ucsc.edu/cgi-bin/hgTracks?hgS_doOtherUser=submit&hgS_otherUserName=mchill&hgS_otherUserSessionName=Qi_Miao_Sox4_Sox11_ChIPseq

Methodology

Replicates	2 replicates per transcription factor (Sox4 and Sox11), and 1 individual input library (IgG).
Sequencing depth	Input IgG; total reads = 70443416, mapped reads = 68635469, % of mapped reads = 97.4%.<br>Sox4 replicate 1; total reads =67069907, mapped reads =65369116, % of mapped reads = 97.5%.<br>Sox11 replicate 1; total reads =68793772, mapped reads =67291787, % of mapped reads = 97.8%.<br>Sox4 replicate 2; total reads =65746871, mapped reads =64027760, % of mapped reads = 97.4%.<br>Sox11 replicate 2; total reads =73102307, mapped reads =71451634, % of mapped reads = 97.7%.<br>All single-end reads, 50 bp in length.
Antibodies	Monoclonal ANTI-FLAG M2 antibody, Sigma, clone M2, F1804
Peak calling parameters	Read mapping: bowtie2 -q -x /bowtie2indexes/mm9/mm9 -U fastq_files -S outputfile.name.sam<br>Peak calling: Homer findPeaks mm9 tag.library -style factor
Data quality	We used a peak calling FDR threshold of 0.001. For Sox4 we identified 2,697 peaks. To remove background peaks we subtracted out regions identified by ENCODE for the mm9 genome using Bedtools subtract, resulting in a total of 2,669 Sox4 peaks. For Sox11 we identified 21,450 peaks, which was cut down to 21,270 peaks after blacklisted regions were removed.
Software	Bowtie2 for mapping reads. HOMER for peak calling and analysis, as well as motif enrichment calculations. We also utilized BEDtools to perform subtraction of the ENCODE blacklisted regions from out peak files (removal of false positives).

Flow Cytometry

Plots

Confirm that:

☐ The axis labels state the marker and fluorochrome used (e.g. CD4-FITC).

☐ The axis scales are clearly visible. Include numbers along axes only for bottom left plot of group (a 'group' is an analysis of identical markers).

☐ All plots are contour plots with outliers or pseudocolor plots.

☐ A numerical value for number of cells or percentage (with statistics) is provided.

Methodology

Sample preparation	*Describe the sample preparation, detailing the biological source of the cells and any tissue processing steps used.*
Instrument	*Identify the instrument used for data collection, specifying make and model number.*
Software	*Describe the software used to collect and analyze the flow cytometry data. For custom code that has been deposited into a community repository, provide accession details.*
Cell population abundance	*Describe the abundance of the relevant cell populations within post-sort fractions, providing details on the purity of the samples and how it was determined.*
Gating strategy	*Describe the gating strategy used for all relevant experiments, specifying the preliminary FSC/SSC gates of the starting cell population, indicating where boundaries between "positive" and "negative" staining cell populations are defined.*

☐ Tick this box to confirm that a figure exemplifying the gating strategy is provided in the Supplementary Information.
